# Preclinical Development of Cell-Based Products: a European Regulatory Science Perspective

**DOI:** 10.1007/s11095-018-2437-y

**Published:** 2018-10-15

**Authors:** James W. McBlane, Parvinder Phul, Michaela Sharpe

**Affiliations:** 1 Licensing Division, Medicines & Healthcare Products Regulatory Agency, 10 South Colonnade, Canary Wharf, London, E14 4PU UK; 2 Nonclinical Safety, Cell and Gene Therapy Catapult, Guy’s Hospital, 12th Floor, Tower Wing B, London, SE1 9RT UK

**Keywords:** biodistribution, cell therapy, regulatory science, toxicity, tumorigenicity

## Abstract

**Purpose:**

This article describes preclinical development of cell-based medicinal products for European markets and discusses European regulatory mechanisms open to developers to aid successful product development. Cell-based medicinal products are diverse, including cells that are autologous or allogeneic, have been genetically modified, or not, or expanded ex vivo, and applied systemically or to an anatomical site different to that of their origin; comments applicable to one product may not be applicable to others, so bespoke development is needed, for all elements - quality, preclinical and clinical.

**Methods:**

After establishing how the product is produced, proof of potential for therapeutic efficacy, and then safety, of the product need to be determined. This includes understanding biodistribution, persistence and toxicity, including potential for malignant transformation. These elements need to be considered in the context of the intended clinical development.

**Results:**

This article describes regulatory mechanisms available to developers to support product development that aim to resolve scientific issues prior to marketing authorization application, to enable patients to have faster access to the product than would otherwise be the case.

**Conclusions:**

Developers are encouraged to be aware of both the scientific issues and regulatory mechanisms to ensure patients can be supplied with these products.

## Introduction

In the development of cell-based medicinal products, data needs to be generated to support progression from testing in animals into testing in patients. Primary pharmacology studies are needed to establish that use of a cell-based therapy could be of benefit in a defined medical setting, and sufficient evidence as to why proposed clinical testing can be considered safe must be presented to regulators and ethical review bodies. This latter element, of reassuring patient safety, especially in initial clinical trials where clinical experience is very limited, includes the ideas of distribution of administered cells, their persistence and considerations of their intended and unintended effects. To place a product on the market, non-clinical testing needs to be complete and demonstrated to be of relevance to the product that is to be used in patients once marketed [1].

This article presents considerations of these elements of development of cell-based medicinal products and also details additional regulatory mechanisms available in the European Union that those undertaking the development of such products can access to aid successful product development.

Cell-based human medicinal products (CBMPs) may contain cells that are autologous, or allogeneic, and may, or may not, include cells that have been genetically modified, or which may, or may not be, combined with a device or with a scaffold or a mesh. This article primarily focusses on medicinal products that contain cells of human origin and of products that are classed as somatic cell therapies; however, tissue engineered products may also contain cells, and the same concepts can apply to these products too. In Europe, only a small number of CBMPs have received regulatory approval and their use is limited [2–4] indicating the possibility that companies may benefit from combining scientific knowledge with regulatory expertise, in order to obtain a marketing authorization.

## Proof of Principle

The objective of primary pharmacology studies is to establish the proof of principle of the CBMP) by use of a suitably robust model to demonstrate the pharmacodynamic activity of the product. These studies aim to support later clinical testing to establish proof of concept. These cell-based products can be complex in their development and so conventional requirements for pharmacological testing that are applied to small, synthetic chemicals are not always appropriate and alternative, more tailored approaches are often more suitable.

Initial proof of principle studies are usually composed of *in vitro* techniques, designed to demonstrate the activity of the CBMP, for example if the proposed role of the CBMP is to restore cell functionality, then the *in vitro* test should be designed in such a way as to demonstrate that normal cell function has been restored and thus provide proof of activity of the CBMP. Likewise, if the proposed effect of administration of the CBMP is to enhance an immune response, then an appropriately designed *in vitro* immunological assay should be used.

For some products, *in vivo* testing in animals is not relevant to predicting effects in humans. For instance, in genetically modified cells targeting a human-specific antigen, administration of these cells to animals would not be expected to result in target engagement, as the target is completely lacking in animals. In these cases, reliance on *in vitro* testing alone may suffice.

A few challenges present themselves when considering the first *in vivo* proof of concept studies [5]. Species difference can be a major obstacle as human cells will likely be rejected by an immunocompetent animal. In an immunocompromised animal, differences in the *in vivo* environment may result in different interactions with tissues and distribution of the cells. In some circumstances, use of animal cells in homologous modelling may be more appropriate, as use of such a product, rather than a human cell-based product, may better indicate the therapeutic potential of a human CBMP in patients, even allowing for such other differences from the human medical setting, such as the method of administration, age of animals and administered cell dose.

It should be noted that the use of homologous animal models may add a level of complexity when extrapolating results to a human therapeutic situation as the product used is not that intended for use in humans.

A successful approach to determining proof of concept is to replicate the target disease or injury, this can be undertaken with the use of animal models of the disease or injury and is potentially useful in determining the activity and safety of the CBMP in order to support progression to clinical trials of the CBMP. These animal models can include induced or spontaneous models of disease, or genetically modified animals (knockouts or transgenics). It is important that models such as these are robustly evaluated for any potential limitations, i.e. variability in results, the absence of historical data with the animal model, adverse health and poor conditioning of disease animal models. Pilot studies are useful in determining the suitability of particular animal models, and it may be necessary to perform more than one model of disease/injury in order to fully characterise the safety and activity of the CBMP.

It is possible that *in vivo* studies are conducted only in small animals, which allows data to be generated from larger numbers of individual animals: use of larger animals typically results in fewer individual animals being used. There is no default expectation that proof of principle should be studies in large animals, and, in fact, the default rests the other way i.e. that studies will be in small animals unless the nature of testing requires that a large animal species be used. In some instances, it is necessary that proof of principle studies be completed in large animals, such as dogs, pigs, goats, sheep or horses. Studies in larger animals have identified the same pathology, or induced similar pathology, as in human medicine and use of prototype medicinal products in these animals has supported development of a human therapeutic [6] Use of larger animals may also be most appropriate when the product is to be given with another component, such as a device, where there is a need to use the intended human device, which may be t impossible in mice or rats. Alternatively, the product may be given using the same injection device as is intended for use in human patients e.g. around areas of infarcted heart muscle tissue [7], where the surgeon who will administer the product to the patient also conducts the animal experiment, in part to develop the necessary skill to deliver the product optimally.

The choice of species used in the pivotal proof-of concept studies should be scientifically rationalised and justified always. A large animal study is not always needed and for some products, *in vivo* proof of principle data can be derived only from testing on rodents. Selection of a first human dose may be based on these *in vivo* animal studies, but it should be born in mid that selection of a dose for first clinical use may also be supported by clinical experience with other CBMPs, where these products are shown to be similar to the specific CBMP in development.

The pivotal proof-of concept studies can include integrated safety end points into their design; this has the advantage of obtaining important safety information related to the administration of the CBMP yet reducing the burden of further animal use for toxicity studies.

In some instances, it may also be possible to base the proof of principle on published data. For example, for chondrocyte type products where a wide level of clinical experience exists, the need to additional proof of concept data may be waived. In these instances, the developer of the CBMP may wish to discuss their approach to establishing the proof of concept with an appropriate competent authority, and in most cases, this would be referred to the CHMP or Committee for Advanced Therapies (CAT) for their scientific advice and expertise. The advice procedure is described in greater detail later in this article. It should be noted that regulatory cell-based guidelines that describe the approaches to examining the pharmacodynamics of CBMPs are available and include, but are not limited to, the CHMP Guideline on human cell-based medicinal products [8].

## Biodistribution / Persistence

Persistence and distribution of these cells is an important feature that must be described in development of a CBMP. This information can have a significant impact on monitoring plans detailed in the protocol for the first human study. Biodistribution studies are particularly necessary when the administration of the product may not be retained to the site of action, for example when the product is implanted locally with the use of a scaffold or membrane but no physical barrier is applied. The potential biodistribution of the implanted CBMP may be demonstrated in a relevant animal model and if so this can, but need not, be integrated into homologous animal models used in the proof of concept studies with the gross and histological examination of several tissues, including the lymph nodes. Study of persistence and biodistribution in studies in larger animals can be included in pivotal proof of principle studies.

Biodistribution studies also seek to provide a basis for designing further studies to establish whether, once implanted, CBMPs could proliferate and differentiate, or would be able to initiate an unintended response (alternative target or effector), or could have potential to accumulate and persist in a particular organ or tissue with possible consequences for long-term safety. Each of these aspects must be addressed. The methods used to track administered cells may pose a technical challenge to the product developer and it is important that methods be developed at an early stage of development in order to accurately track cells *in vivo*.

In most cases, biodistribution studies will be required prior to first human dosing. However, whereas most CBMPs will be given on one occasion only, there are instances where clinical development has changed to test use of a second or third dose, following evidence of rapid cell clearance in humans. In this circumstance, it may be necessary to conduct a repeated dose biodistribution study in animals to support repeated dose clinical testing.

## Toxicity

For CBMPs, the design of conventional toxicity studies, as applied in development of small molecule drugs or of other biological medicinal products and comprising repeat dose toxicity studies of increasing duration in accordance with the intended duration of clinical testing [9–11], is likely to be inappropriate. Instead of considering how to amend such study designs so that they may be applied to a particular cellular therapy, developers should consider the aim of such testing and how this can be achieved most optimally [12–14]. The primary aim is to give reasonable reassurance that use of the clinical CBMP as defined in the clinical protocol will be safe. If there is known to be rapid clearance of administered cells in animals, this can justify short term follow up as longer term follow up will be uninformative for human safety. Often, safety will be combined with assessment of persistence and biodistribution and in some cases, safety assessment can be combined with primary proof of principle studies too.

Although CBMPs share some of the same principle characteristics, it is recognized that cellular therapies are not a homogeneous class of products. In addition, the level of scientific knowledge and clinical experience of a given cellular therapy is highly variable [15]. Given the product-specific attributes of most cellular therapies, a case-by-case, risk-based approach can be taken when designing the nonclinical testing programs. The risk-based approach [16] is based on a series of generic scientific questions that could apply to any cell therapy product. The risk factors are related to the quality, biological activity and clinical application of the cell therapy. By determining the risk for a given therapy, the extent of the nonclinical package can be determined. The factors associated with a given risk are product specific and, in many cases, multifactorial, and each of these needs to be considered as part of the overall assessment [15].

As single administration of a cellular product could result in prolonged exposure in patients, this suggests that duration of follow up in animals may need to be sufficient to support such clinical exposure. However, as noted above, where there is rapid cell clearance in an animal, then it makes no sense to have prolonged follow up; if the cells are known to be gone within 7 days, then it is not necessary to follow up animals for several months, even though prolonged exposure may be expected in humans, as collection of such data from animals is not relevant to predicting effects in humans.

Development of an immune response in normal animals given the human CBMP can be a sufficient reason not to conduct toxicity testing in animals with that product. Alternative evidence to support the safety of patients given the human cell product is needed. This could come from use of the product in normal animals given immunosuppressive drugs, or of use of an immunodeficient animal, or of use of a cell product based on animal cells.

## Tumorigenicity

The tumorigenic potential of cell therapy products can theoretically be influenced by many factors including the differentiation status and proliferation capacity of the cells, genetic modification, the phenotypic plasticity of the cells, the intended clinical location, the long-term survival of the product, genetic and epigenetic changes during culture and genomic alterations during reprogramming (e.g. to form induced pluripotent cells). To date many studies have been run as part of product nonclinical development programmes to address the risk of tumorigenicity; however, as with any study planned to address aspects relating to product safety, the study needs to be relevant [15, 17].

Tumorigenicity studies are largely considered in respect of pluripotent cell derived therapies and the potential for the presence of small numbers of undifferentiated cells within the final product. There is considerable published data on the characteristic of undifferentiated pluripotent cells to form teratomas in immunocompromised animals [18, 19]; indeed, this is a defining characteristic of such cells. The ability of the human immune system, however, to identify pluripotent cells as immunological targets is unknown, but evidence exists that there is T cell reactivity to pluripotency markers such as OCT4 in healthy donors [20], indicating that in a patient with a healthy immune system the risk of tumorigenicity from rare contaminating pluripotent cells may be low. The teratoma studies that have been completed are typically designed to address the issue of contaminating undifferentiated cells; an issue relating to process development and characterisation as well as safety. With questions around the ability of undifferentiated cells to survive in the final product culture conditions, the development of newer *in vitro* methods to characterise the cell product and identify the risk of any contaminating cells and the development of methods to eliminate the undifferentiated cells from the final pluripotent derived cell therapy product (e.g. pluripotent apoptotic agents, stage specific genotoxic agents, activated cell sorting and the use of monoclonal antibodies against undifferentiated stem cell surface markers [21, 22]), consideration is needed as to the appropriateness of routinely running *in vivo* studies to address this specific aspect, or whether the decision to conduct such a study should be a data driven decision in each case. The traditional *in vivo* teratoma assay may also not address the tumorigenic risk of the final differentiated product, an aspect which can be forgotten.

There has also been considerable debate around the propensity of cells and specifically pluripotent cells, that have been maintained in culture for extended periods of time, being taken over by cells carrying genetic abnormalities some of which are highly recurrent [23, 24–26]. For instance, 20% of hPSC lines worldwide carry a gain of a small region of 20q11.21 [23] which has been shown to lead to decreased levels of apoptosis [27, 28]. It is possible that many of the chromosomal changes observed in the pluripotent cell cultures are adaptive and confer a proliferative advantage to the cells but their consequences for the behaviur of particular differentiated cells remain unknown [24]. While some of the genetic aberrations have been associated with cancers, including germ cell tumors, the presence of a mutation per se does not necessarily define the cell as a tumor-causing cell. Even within the general population mutations in the DNA do occur, and for a multitude of reasons, but without causing cancer [29]. It is important that it is understood if any observed DNA mutations are identified as the initiating events causing cancer or whether they act as contributors crucial for the development of a tumor once it has initiated, as this alters the risk profile. The non-randomness seen in the pattern of DNA mutations in cancer cells and also cell therapy products does not mean that these will translate into a causative role [30]. While it is important to capture information around genetic changes within the product the presence per se of a genetic mutation does not necessarily preclude its clinical use.

Somatic cell therapies and in particular mesenchymal stromal cell therapies (MSCs) have been extensively been studied and tested clinically for the treatment of various diseases [30]. Isolated MSCs for example show phenotypic heterogeneity, depending on the origin of the cells and the isolation/manufacturing techniques. Genetic alterations have been reported for MSCs maintained in long term cultures; these include DNA losses and gains, DNA methylation instability, and evidence for telomeric deletions in subpopulations of cells have all been observed during culture to late passage [30]. The risk of tumorigenicity was the focus of a Cell Products Working Party [31] expert meeting which discussed the opportunities and challenges currently faced when MSCs are used as therapeutic products. To date no evidence of tumorigenic potential has been observed following administration of MSC to immunocompromised mice in 3 and 6 month studies [31]. It is possible that the frequency of transformation of somatic cells such as human MSCs is too low to detect overt tumor formation in established rodent models. In addition, in the clinical setting the immunological status of the patient may play a role in determining the risk of tumorigenic potential. An allogeneic therapy will likely be eliminated over time reducing risk.

Genetic modification is the process of modifying or inserting a new genetic sequence into a cell [32] and to date retroviral and lentiviral gene transfer systems are the most commonly employed in the genetic modification of cell therapies since the vectors are capable of sustained high levels of expression and the ability to package large inserts. The insertion of retroviral vectors into the host genome has meant that insertional mutagenesis is a risk for genetically modified cell therapies. Insertional mutagenesis can occur through activation and silencing of genes or dysregulation. Oncogenic activation, has been observed in the clinic following the administration of gamma retrovirally modified hematopoietic stem cells (HSC; [33]), with leukemia or pre-leukemia reported in some patients treated for X-linked Severe Combined Immunodeficiency Disease [34], for Wiskott–Aldrich syndrome [35] and chronic granulomatous disease [36]. In general, the risk of insertional mutagenesis is considered to be related to disease background, cell type to be transduced and vector characteristics [37]. Numerous clinical trials with gamma-retrovirally modified T cells, however, have not yielded evidence for insertional adverse events despite long-term persistence of transduced cells [37] and lentiviral vectors have not yet been associated with insertional oncogenesis, although integration mediated clonal dominance has been reported in one trial [38]. These data suggest that disease background factors and cell-intrinsic mechanisms may modify the risk of insertional mutagenesis.

The risk of tumorigenicity may be assessed by *in vitro* and/or *in vivo* assays; in addition, published data on related products may provide additional supportive information, although the relatedness of the products will need to be determined. For other products, an *in vivo* assessment may be required, although it is strongly advised to discuss the plans with the regulatory authorities in advance of committing to such studies. Where nonclinical *in vivo* studies are run their design needs to take into account assessment of rare events. These studies present a number of challenges including the selection of the optimal animal model, a balance of a feasible group size with statistical power, study duration, dose, and route of administration. In addition, the requirement for special husbandry and care for immunodeficient animals to minimize loss of animals to opportunistic infection exists, particularly for studies that may be of up to one year in duration. Consideration needs to be given to how, or if, these studies can be performed in a manner compliant with Good Laboratory Practice.

Given the variable nature of scientific knowledge and clinical experience with different cell based products, a risk-based approach [17] can be applied. Data provided by the FDA (up to 2014) has shown that across all cell therapies only in 43% of submissions were tumorigenicity assays performed by testing a product directly (*in vitro*/*in vivo*) and that in 57% of cases tumorigenic potential was assumed based on “consideration of product attributes, literature and/or previous clinical experience” [39].

## Regulatory Perspectives

In addition to the scientific considerations discussed above, there are several regulatory perspectives that those undertaking the development of a CBMP should consider [40]. The availability of options to obtain input on product development of regulators is intended to result in eventual wider availability to patients of those products by ensuring that difficult issues are resolved in a manner acceptable to both parties, i.e. product developers and regulators. In contrast to the scientific issues, which are likely to present similar issues in different regions of the world, the considerations below are presented based on experience that is specific to the European Union. However, despite differing regulatory regimes across the world, some of what is considered below may also reflect practice in regions outside the European Union. This article addresses development of cell-based medicinal products but it should be noted that these comments below in A-E are not exclusive to cell-based therapies.

### Expectations for GLP Compliance

In development of medicinal products, studies that are conducted with the intent of characterising product safety are expected to be in compliance with Good Laboratory Practice (GLP) ([41, 42]). However, in some instances, compliance with GLP is not possible: this may be more common with cell therapy products than with other biological products or with small molecule drug products; one example is that testing may take place in academic laboratories which lack capacity for independent quality assurance audit;. typically such studies are done in research facilities where the animals, sometimes with specific genetic abnormalities, happen to be, and these are not facilities able to conduct GLP studies. In circumstances where the generation of preclinical safety data in compliance with GLP is not possible, or where there are existing non-GLP data, such that conducting additional testing would not result in further preclinical safety data of relevance to human use of the product, a claim for a waiver of the expectation for GLP compliance may be accepted by regulatory reviewers [43, 44]. The reason(s) for lack of GLP compliance need to be explained but where there is a justification presented that available and relevant data are considered reliable, lack of compliance with GLP may be acceptable. This should be justified in each case: in these circumstances, it may be prudent for the developer of the product to obtain advice on the acceptability of this approach to regulatory reviewers. This can be done in scientific advice as described next.

### Scientific Advice

In the European context, scientific advice, as it relates to CBMPs, is described in paragraph 23 of Regulation (EC) 1394/2007 [45] and is a procedure that can be applied to any one of, or any combination of, quality, preclinical and clinical data relating to the development of a medicinal product, with the principle aim to give advice on scientific information that may be necessary to demonstrate quality, safety and/or efficacy of the product. This is not limited to cell-based products, but where the advice does relate to a cell-based product, it will be referred to the Committee for Advanced Therapies (CAT) to access relevant expertise. Scientific advice is operated by the European Medicines Agency, the Scientific Advice Working Party (SAWP) and the Committee for Human Medicinal Products (CHMP).

In this procedure, the product developer submits questions and supporting argument for their preferred position and the SAWP and CHMP will review this, and provide a written answer. The developer needs to present sufficient information on the issue(s) for a recommendation to be given and also indicate how it proposes to resolve the issue: Questions can be framed in the form of asking if CHMP agrees with the developer that the information summarised is sufficient to support the developer’s stance. It is key that the developer shows how this information is relevant to the version of its product that is proposed to be put on the market in the European Union.

A consensus European view is given. This procedure involves representatives from all countries of the European Union and offers potential for significant risk-reduction in developing a cell based product because where advice is given that the applicant’s approach is acceptable, it is very unlikely that that issue may be raised as an objection in the review of a marketing authorization application.

In addition, many national competent authorities (the legal term applied to regulatory bodies in each country in the European Union that have responsibility for regulation of medicinal products), offer advice at the national level. Again, these are not limited to non-clinical issues, but these can consider any scientific or regulatory issues relating to product development. National scientific advice is usually in the form of a face-to-face meeting between those developing the product and employees of the national competent authority. It does not carry the same significance in terms of regulatory risk-reduction - in part, this is because each country can only give its own view and there are 27 other countries. Ational scientific advice procedures can be particularly useful in addressing issues relating to specific clinical trials, if the trial is to be conducted in that country.

For more information on scientific advice procedures, see:

http://www.ema.europa.eu/ema/index.jsp?curl=pages/contacts/CHMP/people_listing_000022.jsp

and

http://www.ema.europa.eu/ema/index.jsp?curl=pages/regulation/general/general_content_000049.jsp&mid=WC0b01ac05800229b9.

### EMA Innovation Task Force

Whereas the above scientific advice procedures are focussed on development of a specific medicinal product, the EMA offer the opportunity for early dialogue with developers on product development through its Innovation Task Force. The aim of the EMA Innovation Task Force is to foster innovation in relation to early development programmes, often where the specific product has yet to be manufactured in a form that could be given to human patients. For projects within the scope of the EMA Innovation Task Force, information may be presented on studies relating to the proof of principle (e.g. specificity of antibacterial action in challenge studies in rodents; conditional anti-seizure activity, only in the presence of a second element, itself lacking any intrinsic therapeutic activity) with advice from task force members seeking to find solutions to regulatory challenges posed by the novel nature of the product., where there are difficulties in applying the established models of drug development. Discussions can explore how the developer can optimise development to ensure patient safety in early trials without conducting studies that regulators may not require, but which developers may judge would be requested by regulators. For more information on the EMA Innovation Task Force see:

http://www.ema.europa.eu/ema/index.jsp?curl=pages%2Fregulation%2Fgeneral%2Fgeneral_content_000334.jsp&mid=WC0b01ac05800ba1d9.

### Certification Procedure

Certification is a separate procedure from scientific advice. The certification procedure was created by Article 18 of Regulation (EC) No 1394/2007 [45] and is in place to support development of advanced therapies, which include CBMPs, by small- and medium-sized enterprises: it is not available for products that are not advanced therapies. It is a procedure that aims to offer to those developing such products, and who are eligible, the opportunity to obtain an assessment of their data, as much as is available at the time of seeking certification, so that any issues identified can be addressed prior to making an application for a marketing authorization and so not be reasons to object to approval. The procedure can cover either quality data, alone or quality and non-clinical data but it does not extend to review of clinical data, although the review is conducted in light of a general understanding of the intended therapeutic use. Where quality data have been certified alone in a previous procedure, non-clinical data can be certified with updated quality data. It is operated by the European Medicines Agency and the Committee for Advanced Therapies (CAT) [46].

The certification procedure involves the scientific evaluation of data submitted from the perspective of whether these data would meet the standards that apply for evaluating a marketing authorization application.

The certification procedure operates thus. After receipt of the application, one member of the CAT is assigned as the CAT Rapporteur and undertakes assessment of data submitted and prepares a report, which is reviewed by a second member of the CAT, acting in the capacity of Peer Reviewer. The position then agreed is put to the full committee who discuss the proposals, both those presented by the applicant, and the responses proposed by the CAT Rapporteur and a final position is agreed by CAT and the Rapporteur’s report is amended as needed to reflect this final position. The CAT may recommend to the EMA to certify the data reviewed, which confirms the extent to which the data meet expectations applied in licensing decisions, or the CAT may identify deficiencies in the dataset that should be resolved by the time of making a marketing authorization application. This procedure takes 90 days and it is voluntary.

For more information on the certification procedure, see: http://www.ema.europa.eu/ema/index.jsp?curl=pages/regulation/general/general_content_000300.jsp&mid=WC0b01ac058007f4bd.

### Prime Scheme

The PRIME (PRIority MEdicine) scheme exists to facilitate approval of products that target an unmet medical need. This, too, is voluntary and the scheme is open to any type of medicinal product that may address an unmet need. The scheme aims support engagement between regulators and product developers so that appropriate studies are done and when the marketing authorization application is received, to allow fast evaluation, all with the aim that patients can access the product sooner than would otherwise be the case.

Initially, the developer should form its own view as to why its product meets the definition of a priority medicine, as having potential to meet an unmet medical need: at least, early clinical data are expected. The Scientific Advice Working Party (SAWP) reviews applications for PRIME designation, for CBMPs taking into account considerations from the Committee for Advanced Therapies (CAT) and where granted, there is a Rapporteur appointed who supports development of the product with regular meetings with its developers. The aim is to ensure that the applicant understands the expectations of regulators when designing studies on which the decision to approve, or not, will be based.

For more information on the PRIME scheme see: http://www.ema.europa.eu/ema/index.jsp?curl=pages/regulation/general/general_content_000660.jsp.

## Conclusion

In this article, the authors have given a short summary of the scientific issues facing product developers on the preclinical development of a cell based medicinal product and also explained some of the existing mechanisms for accessing support for product development from European regulatory authorities. The scope of product type that is included in the term cell-based therapy is broad and making specific comments applicable to one product may not be useful if applied to another product: products that are fundamentally different may require different approaches to their development. However, the aims of preclinical development remain: to provide evidence that the product may have beneficial effects, if used in a specific manner in a defined medical setting and to demonstrate that it is safe to test in human patients in the manner proposed. There have been few newly licensed CBMPs in the last 10 years, when the relevant legislation came into force in Europe [42], and one implication of this is that product development is difficult. For the most part, preclinical development has not been the cause of a failure to license products. The key element is to demonstrate that patient benefit is due to the product; the preclinical issues described here need to be addressed to get to that point.

## Abbreviations

CAT: Committee for Advanced Therapies
CBMP: cell based medicinal product
CHMP: Committee for Human Medicinal Products
CPWP: Cell Products Working Party
DNA: deoxyribonucleic acid
EC: European Commission
EMA: European Medicines Agency
FDA: Food and Drug Administration
GLP: Good Laboratory Practice
hPSC: human pluripotent stem cells
HSC: hematopoietic stem cells
ICH: International Conference on Harmonisation
MSC: mesenchymal stromal cells
SAWP: Scientific Advice Working Party
SWP: Safety Working Party

## References

[1] McBlane JW (2015). Preclinical safety testing for cell-based products using animals. Biologicals.

[2] Aiuti A, Roncarolo MG, Naldini L (2017). Gene therapy for ADA-SCID, the first marketing approval of an ex vivo gene therapy in Europe: paving the road for the next generation of advanced therapy medicinal products. EMBO Mol Med.

[3] McBlane JW (2015). Regulatory landscape for cell therapy – EU view. Biologicals.

[4] Yano K, Watanabe N, Tsuyuki K, Ikawa T, Kasanuki H, Yamato M (2015). Regulatory approval for autologous human cells and tissue products in the United States, the European Union, and Japan. Regen Ther.

[5] Vestergaard HT, D'Apote L, Schneider CK, Herberts C (2013). The evolution of nonclinical regulatory science: advanced therapy medicinal products as a paradigm. Mol Ther.

[6] Dorotka R, Bindreiter U, Macfelda K, Windberger U, Nehrer S (2005). Marrow stimulation and chondrocyte transplantation using a collagen matrix for cartilage repair. Osteoarthr Cartil.

[7] Schüssler-Lenz M, Beneu C, Menezes-Ferreira M, Jekerle V, Bartunek J, Chamuleau S, Celis P, Doevendans P, O’Donovan M, Hill J, Hystad M, Jovinge S, Kyselovič J, Lipnik-Stangelj M, Maciulaitis R, Prasad K, Samuel A, Tenhunen O, Tonn T, Rosano G, Zeiher A, Salmikangas P (2016). Cell-based therapies for cardiac repair: a meeting report on scientific observations and European regulatory viewpoints. Eur Heart J.

[8] CHMP/410869/2006 Guideline on Human Cell-Based Medicinal Products.

[9] ICHS6(R1) Preclinical safety evaluation of biotechnology-derived pharmaceuticals. 10.1038/nrd822 12119749

[10] ICHM3(R2) Non-clinical safety studies for the conduct of human clinical trials and marketing authorisation for pharmaceuticals. 20349552

[11] CHMP/SWP/1042/99 (R1) Guideline on repeated dose toxicity.

[12] Blaich G, Baumann A, Kronenberg S, de haan L, Ulrich P, Richter WF, Tibbitts J, Chivers S, Tarcsa E, Caldwell R, Crameri F (2016). Non-clinical safety evaluation of biotherapeutics – challenges, opportunities and new insights. Regul Toxicol Pharmacol.

[13] Goldring CE, Duffy PA, Benvenisty N, Andrews PW, Ben-David U, Eakins R, French N, Hanley NA, Kelly L, Kitteringham NR, Kurth J, Ladenheim D, Laverty H, McBlane J, Narayanan G, Patel S, Reinhardt J, Rossi A, Sharpe M, Park BK (2011). Assessing the safety of stem cells. Cell Stem Cell.

[14] Hayakawa T, Aoi T, Bravery C, Hoogendoorn K, Knezevic I, Koga J, Maeda D, Matsuyama A, McBlane J, Morio T, Petricciani J, Rao M, Ridgway A, Sato D, Sato Y, Stacey G, Sakamoto N, Trouvin JH, Umezawa A, Yamato M, Yano K, Yokote H, Yoshimatsu K, Zorzi-Morre P (2015). Report of the international conference on regulatory endeavors towards the sound development of human cell therapy products. Biologicals.

[15] Bailey AM (2013). Balancing tissue and tumor formation in regenerative medicine. Sci Transl Med.

[16] Sharpe Michaela, Leoni Giulia, Barry Jacqueline, Schutte Rindi, Mount Natalie (2016). Nonclinical Studies for Cell-Based Medicines. Guide to Cell Therapy GxP.

[17] Kooijman M, van Meer PJ, Gispen-de Wied CC, Moors EH, Hekkert MP, Schellekens H (2013). The risk-based approach to ATMP development - generally accepted by regulators but infrequently used by companies. Regul Toxicol Pharmacol.

[18] Thomson JA (1998). Embryonic stem cell lines derived from human blastocysts. Science 1999.

[19] Blum B, Benvenisty N (2009). The tumorigenicity of diploid and aneuploid human pluripotent stem cells. Cell Cycle.

[20] Dhodapkar MV (2002). Antigen-bearing immature dendritic cells induce peptide-specific CD8+ regulatory T cells *in vivo* in humans. Blood.

[21] Sharpe ME, Morton D, Rossi A (2012). Nonclinical safety strategies for stem cell therapies. Toxicol Appl Pharmacol.

[22] Conesa C, Doss MX, Antzelevitch C, Sachinidis A, Sancho J, Carrodeguas JA (2012). Identification of specific pluripotent stem cell death-inducing small molecules by chemical screening. Stem Cell Rev.

[23] Lee MO, Moon SH, Jeong HC, Yi JY, Lee TH, Shim SH, Rhee YH, Lee SH, Oh SJ, Lee MY, Han MJ, Cho YS, Chung HM, Kim KS, Cha HJ (2013). Inhibition of pluripotent stem cell-derived teratoma formation by small molecules. Proc Natl Acad Sci U S A.

[24] Amps K, Andrews PW, Anyfantis G, Armstrong L, Avery S, Baharvand H, Baker J, Baker D, Munoz MB, Beil S, Benvenisty N, Ben-Yosef D, Biancotti JC, Bosman A, Brena RM, Brison D, Caisander G, Camarasa MV, Chen J, Chiao E, Choi YM, Choo AB, Collins D, Colman A, Crook JM, Daley GQ, Dalton A, De Sousa PA, Denning C, Downie J, Dvorak P, Montgomery KD, Feki A, Ford A, Fox V, Fraga AM, Frumkin T, Ge L, Gokhale PJ, Golan-Lev T, Gourabi H, Gropp M, Lu G, Hampl A, Harron K, Healy L, Herath W, Holm F, Hovatta O, Hyllner J, Inamdar MS, Irwanto AK, Ishii T, Jaconi M, Jin Y, Kimber S, Kiselev S, Knowles BB, Kopper O, Kukharenko V, Kuliev A, Lagarkova MA, Laird PW, Lako M, Laslett AL, Lavon N, Lee DR, Lee JE, Li C, Lim LS, Ludwig TE, Ma Y, Maltby E, Mateizel I, Mayshar Y, Mileikovsky M, Minger SL, Miyazaki T, Moon SY, Moore H, Mummery C, Nagy A, Nakatsuji N, Narwani K, Oh SK, Oh SK, Olson C, Otonkoski T, Pan F, Park IH, Pells S, Pera MF, Pereira LV, Qi O, Raj GS, Reubinoff B, Robins A, Robson P, Rossant J, Salekdeh GH, Schulz TC, Sermon K, Mohamed JS, Shen H, Sherrer E, Sidhu K, Sivarajah S, Skottman H, Spits C, Stacey GN, Strehl R, Strelchenko N, Suemori H, Sun B, Suuronen R, Takahashi K, Tuuri T, Venu P, Verlinsky Y, Ward-van Oostwaard D, Weisenberger DJ, Wu Y, Yamanaka S, Young L, Zhou Q (2011). Screening ethnically diverse human embryonic stem cells identifies a chromosome 20 minimal amplicon conferring growth advantage. Nat Biotechnol.

[25] Baker D, Hirst AJ, Gokhale PJ, Juarez MA, Williams S, Wheeler M, Bean K, Allison TF, Moore HD, Andrews PW, Barbaric I (2016). Detecting genetic mosaicism in cultures of human pluripotent stem cells. Stem Cell Reports.

[26] Jacobs K, Zambelli F, Mertzanidou A, Smolders I, Geens M, Nguyen HT, Barbe L, Sermon K, Spits C (2016). Higher-density culture in human embryonic stem cells results in DNA damage and genome instability. Stem Cell Reports.

[27] Yoshihara M, Hayashizaki Y, Murakawa Y (2017). Genomic instability of iPSCs: challenges towards their clinical applications. Stem Cell Rev.

[28] Avery S, Hirst AJ, Baker D, Lim CY, Alagaratnam S, Skotheim RI, Lothe RA, Pera MF, Colman A, Robson P, Andrews PW, Knowles BB (2013). BCL-XL mediates the strong selective advantage of a 20q11.21 amplification commonly found in human embryonic stem cell cultures. Stem Cell Reports..

[29] Nguyen HT, Geens M, Mertzanidou A, Jacobs K, Heirman C, Breckpot K, Spits C (2014). Gain of 20q11.21 in human embryonic stem cells improves cell survival by increased expression of Bcl-xL. Mol Hum Reprod.

[30] Adjiri A (2017). DNA mutations may not be the cause of cancer. Oncol Ther.

[31] Barkholt L, Flory E, Jekerle V, Lucas-Samuel S, Ahnert P, Bisset L, Büscher D, Fibbe W, Foussat A, Kwa M, Lantz O, Mačiulaitis R, Palomäki T, Schneider CK, Sensebé L, Tachdjian G, Tarte K, Tosca L, Salmikangas P (2013). Risk of tumorigenicity in mesenchymal stromal cell-based therapies - bridging scientific observations and regulatory viewpoints. Cytotherapy.

[32] Phillips MI, Tang YL (2008). Genetic modification of stem cells for transplantation. Adv Drug Deliv Rev.

[33] Ferrua Francesca, Aiuti Alessandro (2017). Twenty-Five Years of Gene Therapy for ADA-SCID: From Bubble Babies to an Approved Drug. Human Gene Therapy.

[34] Hacein-Bey-Abina S, Garrigue A, Wang GP, Soulier J, Lim A, Morillon E, Clappier E, Caccavelli L, Delabesse E, Beldjord K, Asnafi V, MacIntyere E, Dal Cortivo L, Radford I, Brousse N, Sigaux F, Moshous D, Hauer J, Borkhardt A, Belohradsky BH, Wintergesrt U, Velez MC, Leiva L, Sorensen R, Wulffraat N, Blanche S, Bushman FD, Fischer A, Cavazzana-Calvo M (2008). Insertional oncogenesis in 4 patiens after retrovirus-mediated gene therapy of SCID-X1. J Clin Invest.

[35] Braun CJ, Boztug K, Paruzynski A, Witzel M, Schwarzer A, Rothe M, Modlich U, Beier R, Gohring G, Steinemann D, Fronza R, Ball CR, Haemmerle R, Naundorf S, Kuhlcke K, Rose M, Fraser C, Mathias L, Ferrari R, Abboud MR, Al-Herz W, Kondratenko I, Marodi L, Glimm H, Schlegelberger B, Schambach A, Albert MH, Schmidt M, von Kalle C, Klein C (2014). Gene therapy for Wiskott-Aldrich syndrome - long-term efficacy and genotoxicity. Sci Transl Med.

[36] Ott MG, Schmidt M, Schwarzwaelder K, Stein S, Siler U, Koehl U, Glimm H, Kuhlcke K, Schilz A, Kunkel H, Naundorf S, Brinkmann A, Deichmann A, Fischer M, Ball C, Pilz I, Dunbar C, Du Y, Jenkins NA, Copeland NG, Luthi U, Hassan M, Thrasher AJ, Hoelzer D, von Kalle C, Seger R, Grez M (2006). Correction of X-linked chronic granulomatous disease by gene therapy, augmented by insertional activation of MDS1-EVI1, PRDM16 or SETBP1. Nat Med.

[37] Persons DA, Baum C (2011). Solving the problem of gamma-retroviral vectors containing long terminal repeats. Mol Ther.

[38] Cavazzana-Calvo M, Payen E, Negre O, Wang G, Hehir K, Fusil F, Down J, Denaro M, Brady T, Westerman K, Cavallesco R, Gillet-Legrand B, Caccavelli L, Sgarra R, Maouche-Chretien L, Bernaudin F, Girot R, Dorazio R, Mulder GJ, Polack A, Bank A, Soulier J, Larghero J, Kabbara N, Dalle B, Gourmel B, Socie G, Chretien S, Cartier N, Aubourg P, Fischer A, Cornetta K, Galacteros F, Beuzard Y, Gluckman E, Bushman F, Hacein-Bey-Abina S, Leboulch P. Transfusion independence and HMGA2 activation after gene therapy of human beta-thalassaemia. Nature 2010 467;(7313):318–322. 10.1038/nature09328 PMC3355472 20844535

[39] Bailey AM, Mendicino M, Au P (2014). An FDA perspective on preclinical development of cell-based regenerative medicine products. Nat Biotechnol.

[40] Flory E, Reinhardt J (2013). European regulatory tools for advanced therapy medicinal products. Transfus Med Hemother.

[41] Directive 2004/10/EC of the European Parliament and of the Council of 11 February 2004 on the harmonisation of laws, regulations and administrative provisions relating to the application of the principles of good laboratory practice and the verification of their application for tests on chemical substances.

[42] Regulation (EC) 1272/2008 of the European Parliament and of the Council of 16 December 2008 on classification, labelling and packaging of substances and mixtures, amending and repealing Directives 67/548/EEC and 1999/45/EC, and amending Regulation (EC) No 1907/2006.

[43] Volk SW, Theoret C (2013). Translating stem cell therapies: the role of companion animals in regenerative medicine. Wound Repair Regen.

[44] Cavagnaro J, Silva Lima B (2015). Regulatory acceptance of animal models of disease to support clinical trials of medicines and advanced therapy medicinal products. Eur J Pharmacol.

[45] Regulation (EC) 1394/2007 of the European Parliament and of the Council of 13 November 2007 on Advanced Therapy Medicinal Products and Amending Directive 2001/83/EC and Regulation (EC) No 726/2004.

[46] EMA/CAT/486831/2008/corr Guideline on the Minimum Quality and Non-Clinical Data for Certification of Advanced Therapy Medicinal Products.

